# Multi-color imaging of magnetic Co/Pt heterostructures

**DOI:** 10.1063/1.4976004

**Published:** 2017-02-16

**Authors:** Felix Willems, Clemens von Korff Schmising, David Weder, Christian M. Günther, Michael Schneider, Bastian Pfau, Sven Meise, Erik Guehrs, Jan Geilhufe, Alaa El Din Merhe, Emmanuelle Jal, Boris Vodungbo, Jan Lüning, Benoit Mahieu, Flavio Capotondi, Emanuele Pedersoli, David Gauthier, Michele Manfredda, Stefan Eisebitt

**Affiliations:** 1 Max-Born-Institute Berlin, 12489 Berlin, Germany; 2 Institut für Optik und Atomare Physik, Technische Universität Berlin, 10623 Berlin, Germany; 3 Sorbonne Universités, UPMC Université Paris 06, UMR 7614, LCPMR, 75005 Paris, France; 4 CNRS, UMR 7614, LCPMR, 75005 Paris, France; 5 Laboratoire d'Optique Appliquée, ENSTA ParisTech, CNRS, Ecole Polytechnique, Université Paris-Saclay, 828 boulevard des Maréchaux, 91762 Palaiseau Cedex, France; 6 Elettra-Sincrotrone Trieste, 34149 Basovizza, Trieste, Italy

## Abstract

We present an element specific and spatially resolved view of magnetic domains in Co/Pt heterostructures in the extreme ultraviolet spectral range. Resonant small-angle scattering and coherent imaging with Fourier-transform holography reveal nanoscale magnetic domain networks via magnetic dichroism of Co at the M_2,3_ edges as well as via strong dichroic signals at the O_2,3_ and N_6,7_ edges of Pt. We demonstrate for the first time simultaneous, two-color coherent imaging at a free-electron laser facility paving the way for a direct real space access to ultrafast magnetization dynamics in complex multicomponent material systems.

## I. INTRODUCTION

Magnetic systems with Co/Pt interfaces exhibit a wealth of intriguing phenomena based on strong spin orbit interaction. Some recent examples include the control of domain wall motion arising from Dzyaloshinskii-Moriya interaction and spin Hall currents,^1^ room temperature dynamics of skyrmions in a magnetic racetrack geometry^2^ and ultrafast, all-optical control of electric currents in ferromagnetic heterostructures^3^ and present promising new opportunities for spintronic devices based on Co/Pt sample systems. Furthermore, femtosecond optical excitation of bilayers of ferromagnetic and nonferromagnetic layers have been shown to induce an efficient spin-to-charge conversion via the inverse Hall effect^4^ and have led to efficient ultrabroadband emitters of terahertz radiation.^5^ Finally, all-optical helicity-dependent switching in the technologically important class of Co/Pt multilayers and FePt granular thin films^6^ has triggered an intense debate discussing the responsible microscopic processes.^7–9^ In particular, the hypothesis that the optically induced switching is triggered by an initial stochastic nucleation process in form of mesoscopic magnetic domain structures^10,11^ calls for novel experimental techniques that give a direct and simultaneous access to the element specific magnetization with nanometer spatial and femtosecond temporal resolution.

Novel light sources like high harmonic generation (HHG) and free electron lasers (FELs) generate a brilliant radiation covering the spectral range from the extreme ultraviolet (XUV) to the soft X-ray region with unique properties regarding its ultrashort temporal pulse structure for femtosecond time resolution, its tunable photon energies for element-selective spectroscopy and its high degree of spatial coherence for nanoscale imaging techniques. Additionally, laser generated high harmonic spectra and novel two-color schemes at free electron laser facilities^12,13^ allow simultaneous probing of different elements of complex materials.

In this contribution, we present a magnetic small-angle scattering (SAXS) and Fourier transform holography (FTH) experiments of Co/Pt heterostructures in the XUV spectral range. Strong magnetic scattering cross sections exist at both the Co M_2,3_ edges as well at the O_2,3_ and N_6,7_ edges of Pt, leading to corresponding bright 1st order diffraction in SAXS and high-contrast and high-resolution real space images in FTH. These results allow us to design and carry out the first two-color coherent imaging experiment at the free-electron laser facility FERMI, where a single hologram encodes the real space information of the magnetic domain network stemming from Co and Pt.

## II. MAGNETIC RESONANT SMALL-ANGLE SCATTERING

The performed magnetic resonant small-angle scattering experiment serves to determine the amplitude of the magnetic scattering cross section as a function of energy as well to determine the average length scale of the magnetic nanostructure. This allows a fast benchmark of the sample system and identifies the optimal energy range for the increasingly complex coherent single- and two-color imaging experiments.

**FIG. 1.**
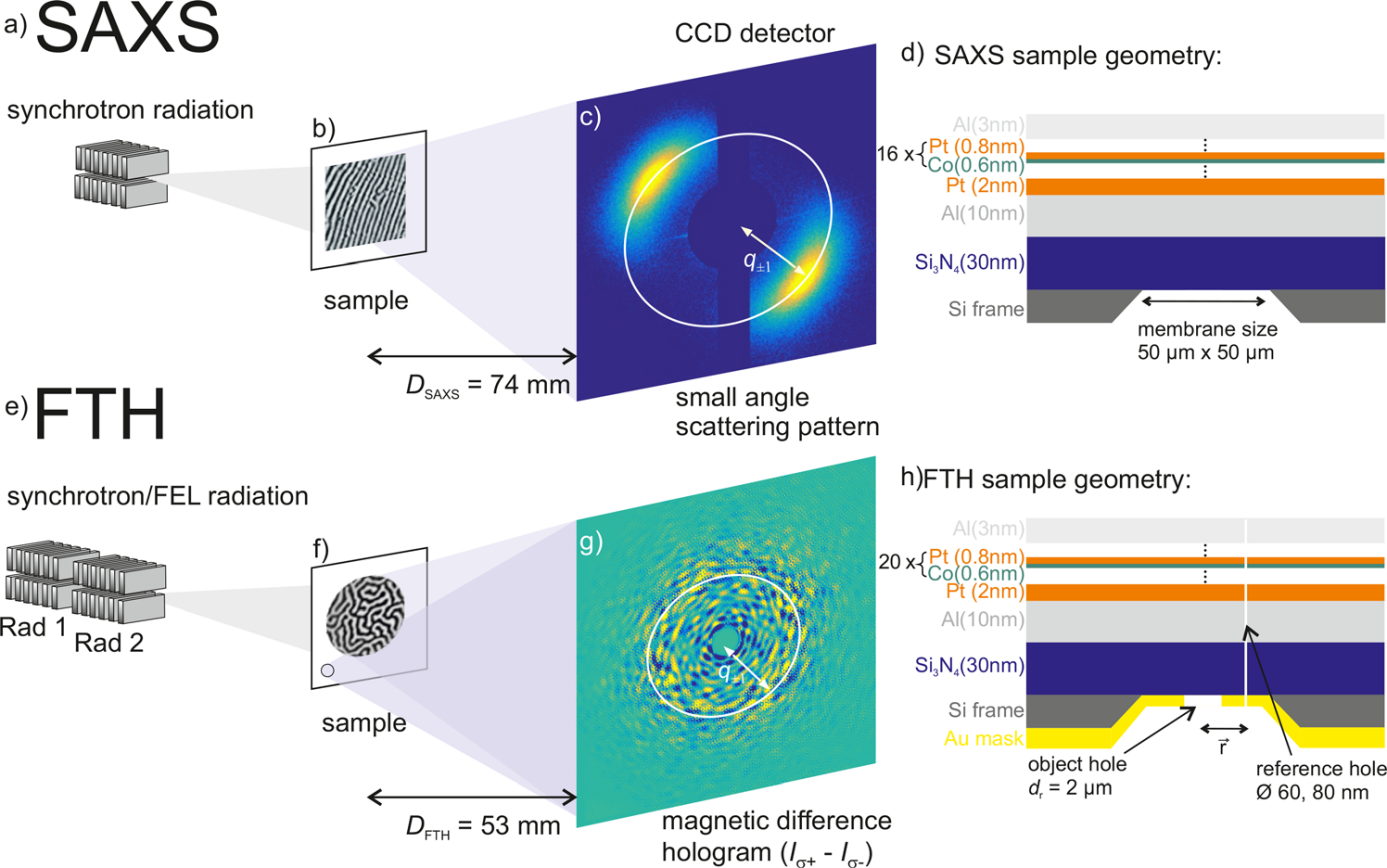
Schematic of the experimental setup for (a) resonant small-angle X-ray scattering (SAXS) and (e) magnetic Fourier transform holography (FTH). In the SAXS experiment, the magnetic domains are aligned in stripes (b) and lead to bright first order scattering peaks centered at a momentum transfer *±q* (c). In the FTH experiment, the magnetic domains exhibit a labyrinth network (f) leading to an isotropic magnetic small angle scattering pattern in the difference image between left and right circular polarization (*I*_σ+_−*I*_σ−_) (g). The corresponding sample compositions and geometries are shown on the right hand side ((d) and (h)).

The experimental setup of the SAXS experiment is schematically shown in Figure 1(a). The energy dependent small-angle scattering experiment was performed at the synchrotron facility BESSY II at the undulator beamline UE112-PGM.^14^ The number of photons in the energy range between 35 eV and 80 eV is on the order of 10^13 ^ph/s; a monochromator yields a maximal energy resolution of *E*/Δ*E* > 20.000. The Co/Pt multilayer (Fig. 1(d)), with a composition of Al(10)/Pt(2)/[Co(0.6/Pt(0.8))]_16_/Al(3) nm, and out-of-plane anisotropy was deposited on a Si_3_N_4_ membrane (50 *μ*m × 50 *μ*m × 30 nm) by magnetron sputtering. Prior to the small-angle scattering experiment, the magnetic domains were aligned to form a stripe geometry by applying an oscillating, successively decreasing in-plane external magnetic field (Fig. 1(b)).^15^ The alternating magnetized domains have an opposite dichroic index of refraction at core-hole transitions such that the sample acts as a magnetic diffraction grating.^16,17^ The advantage of a stripe pattern for magnetic SAXS experiments is manifold: first of all it leads to well defined diffraction spots in comparison to a spread out ring diffraction pattern predicted for an isotropic labyrinth domain network. This leads to an improved signal to noise ratio, without influencing the energy dependence of the magnetic scattering intensity. Furthermore, the two unused quadrants of the charged-coupled device (CCD) detector allow to simultaneously collect scattering from additional grating structures integrated into the sample substrate for XUV beam intensity monitoring.^18^ Finally, we avoid an overlap of the scattering pattern with the beam stop. The sample was placed close to the focus of the XUV beam, and the scattering pattern was recorded with a back-illuminated charged-coupled device (CCD) placed *D*_SAXS_= 74 mm downstream of the sample, sufficiently close to detect the first order diffraction peaks for the smallest energy at 35 eV. The direct beam and charge scattering of the membrane edges were blocked by a beam stop. The polarization of the XUV radiation was set to negative circular helicity. We set the integration time to 1 s to avoid saturation of the CCD detector and accumulated 4 images for each photon energy between 35 eV to 80 eV in 0.5 eV steps.

**FIG. 2.**
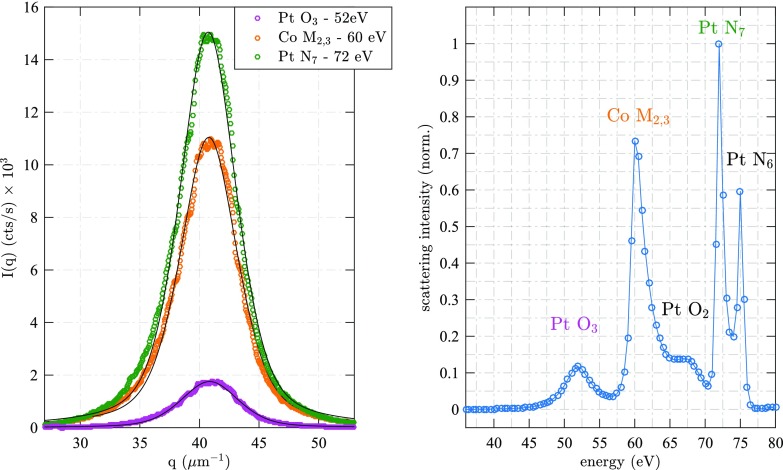
(a) Azimuthally integrated small-angle XUV scattering of an aligned magnetic domain network resonant at the Co M_2,3_ edge at *E*_ph_ = 60 eV, Pt O_3_ and N_6_ edge at *E*_Ph_ = 52 and 72 eV, respectively, as a function of the scattering vector *q*. The solid lines are non-linear least square fits to a pseudo-Voigt function centered at *q*_±1_ = 41 *μ*m^−1^ with a full width at half maximum of *Δq*_±1_ = 6.6 *μ*m^−1^. (b) Energy spectrum of the total number of scattered photons determined by calculating the area of the corresponding pseudo-Voigt functions. We identify the pronounced magnetic dichroism at the Co M_2,3_ edge as well at the Pt O_2,3_ and N_6,7_ transitions. Note that the scattering intensity at the Pt N_7_ edges is spectrally very narrow and that its peak value is significantly larger than at the Co M_2,3_ edge. The solid line is a guide to the eye.

The scattering pattern for a photon energy of *E*_ph_ = 60 eV, resonant at the Co M_2,3_ edge, is shown in Figure 1(c) and exhibits two bright spots indicating that the magnetic domains are indeed in a well aligned domain state. In Figure 2(a), we plot the azimuthally integrated scattering intensity for XUV photon energies of 52 eV, 60 eV, and 72 eV as a function of the momentum transfer *q*, which is calculated as

$$q=\frac{4\pi }{\lambda }\text{sin}(\Theta )=\frac{4\pi \;E_{Ph}}{hc}\text{sin}(\frac{1}{2}\text{atan}\left(\frac{d_{pix}\cdot r}{D_{SAXS}}\right)).$$ (1)

*E*_Ph_(*λ*) is the XUV photon energy (wavelength), θ the scattering angle, *d*_pix_ the side length of a CCD pixel (13.5 *μ*m), and *r* the radius in pixels along which each azimuthal integration is performed. The *q* values are corrected for the small deviation of the planar CCD detector from a sphere to the absolute momentum transfer in reciprocal space. The profiles are well described by a pseudo-Voigt profile, a non-linear least square fit (solid lines in Figure 2(a)) determines a constant center at *q*_±1_ = 40.9 *μ*m^−1^ with a full width at half maximum of *Δq*_±1_ = 6.6 *μ*m^−1^. These values are constant over the entire measured energy range with a standard deviation of 0.1 *μ*m^−1^. This corresponds to an average magnetic domain periodicity of *d*_dw_ = 2π/*q*_±1_= (154 ± 1) nm. The area of the pseudo-Voigt function is a measure for the total scattering intensity and is shown in Figure 2(b) as a function of the photon energy. We identify 5 distinct intensity maxima which we can assign to the following magnetically dichroic resonances: Co M_2,3_ (3*p*_1/2,3/2_ → 3*d*) centered around 60 eV, Pt O_2,3_ (5*p*_1/2,3/2_ → 3*d*) at 66 eV and 52 eV and Pt N_6,7_ (4*f*_5/2,7/2_ → 5*d*) at 75 eV and 72 eV, respectively. These values are in qualitative agreement with previously measured magnetic circular dichroism (XMCD) spectra.^19–21^ However, one needs to keep in mind that the magnetic domains act as both a magnetic phase and an absorption grating. Note that the signal at the Pt N_6_ edge has a very narrow spectral width below our energy step width of 0.5 eV and significantly exceeds the scattering intensity of the Co M_2,3_ edge. Since the resonantly scattered intensity is proportional to the square of the magnetic structure factor,^16,22^ this small-angle XUV scattering experiment acts as a very sensitive probe for the magnetization with element specificity and access to nanometer spatial resolution. In the present case, we measure the lateral spatial profiles of the magnetization in the Co layers as well as the induced magnetization in Pt; the energy independent momentum transfer *q*_±1_ clearly indicates a laterally homogeneous magnetization of the entire multilayer. Spatial separation of the scattering peaks in a two- or multi-color experiment for simultaneous element specificity at Co and Pt can be achieved by adapting the sample detector distance *D*_SAXS_. Furthermore, it is noteworthy that the Pt N_6_ resonance is below the Al L-edge, which allows the use of Al metallic filters against visible and infrared radiation for time resolved, optical pump-XUV probe studies using free electron laser or high harmonic radiation.^17,23^ Because the induced magnetization of Pt is known to be confined to a few atomic layers at the Co/Pt boundary,^19,24^ envisioned time resolved experiments will hence not only track the lateral spatial magnetization profiles after ultrafast laser excitation, but will also give a detailed view on the physics of interface magnetism.^21^

## III. FOURIER TRANSFORM HOLOGRAPHY

Fourier-transform holography encodes the real-space information of the magnetic nanostructure by interference of the magnetic small-angle scattering stemming from the object with a known reference wave. Because of this direct connection between SAXS and FTH (cf. Figures 1(c) and 1(g)), we take the measured energy spectrum of the SAXS intensity (Figure 2(b)) to infer the optimal energy range for which we can image the element specific magnetization of Co and Pt with a maximal signal to noise ratio.

The experimental geometry for the coherent imaging experiments via Fourier transform holography is shown in Figure 1(e). The measurement was also performed at the undulator beamline UE112-PGM^14^ of the synchrotron facility BESSY II (HZB). The sample was placed approximately 100 mm behind the focus, where we confirmed a high degree of transversal coherence via a Young double slit interference experiment.^25^ We determined a coherence |*μ*_12_| > 90% for a slit separation of 18 *μ*m, significantly exceeding the object-reference distance of $\left|\vec{r}\right|=5$ *μ*m. The holographic mask was manufactured in a standard transmission configuration:^26–28^ A silicon nitride membrane (thickness 30 nm) supported by a silicon frame acted as a substrate. After evaporation of an XUV opaque gold film (thickness 250 nm, maximal transmission <10^−8^ for energies between 57 eV and 76 eV), the field of view is defined by drilling a circular object hole with a diameter of $d_{r}=2$ *μ*m via ion-beam lithography. Subsequently, the magnetic multilayer film Al(10)/Pt(2)/[Co(0.6)/Pt(0.8)]_20_/Al(3) nm was deposited via magnetron sputtering, and finally 5 reference holes with 60 nm and 80 nm diameter were added (Figure 1(h)). The coherent scattering from the object and the reference holes interfere with the CCD camera placed *D*_FTH_ = 53 mm behind the sample and form the intensity hologram. Due to the limited dynamic range of the camera, we block the direct beam by a circular beam stop. We recorded five holograms for positive, σ^+^, and negative, σ^–^, helicity for XUV photon energies ranging from 57 eV to 65 eV and from 71 eV to 75.5 eV in 0.5 eV steps. The integration time for a single hologram varied from 20 to 25 s making full use of the dynamic range of the detector. The difference hologram (*I*_σ+_ − *I*_σ+_) contains only magnetic contributions, an example for *E*_Ph_ = 60 eV is shown in Figure 1(g). The hologram exhibits pronounced magnetic speckles in the small-angle scattering signal due to the coherent illumination of the masked sample area. In addition, strong intensity fringes from the object–reference interference appear with a period of approximately 16 pixels extending all the way to the edge of the detector. The selected energy range around the Co M and Pt N edges for the imaging experiment was chosen according to the SAXS measurement (Figure 2(b)) combining the maximal scattering strength as well as the best element specificity. Furthermore the Pt N edges are at a significantly smaller wavelength compared to the Pt O_3_ edge and will therefore yield a superior spatial resolution. Specifically, the experimental geometry with *D*_FTH_ = 53 mm results in a maximum recorded wave vector transfer *q*_max_ = 74 *μ*m^−1^ at 57 eV and 97 *μ*m^−1^ at 75.5 eV (cf. Equation (1)), corresponding to encoded spatial frequencies of *d*_re_ = 2π/q = 85 nm and 65 nm, respectively. Note, that in our experiment, the spatial resolution is also limited by the size of the reference hole and is estimated to be on the order of 80 nm (see below).

**FIG. 3.**
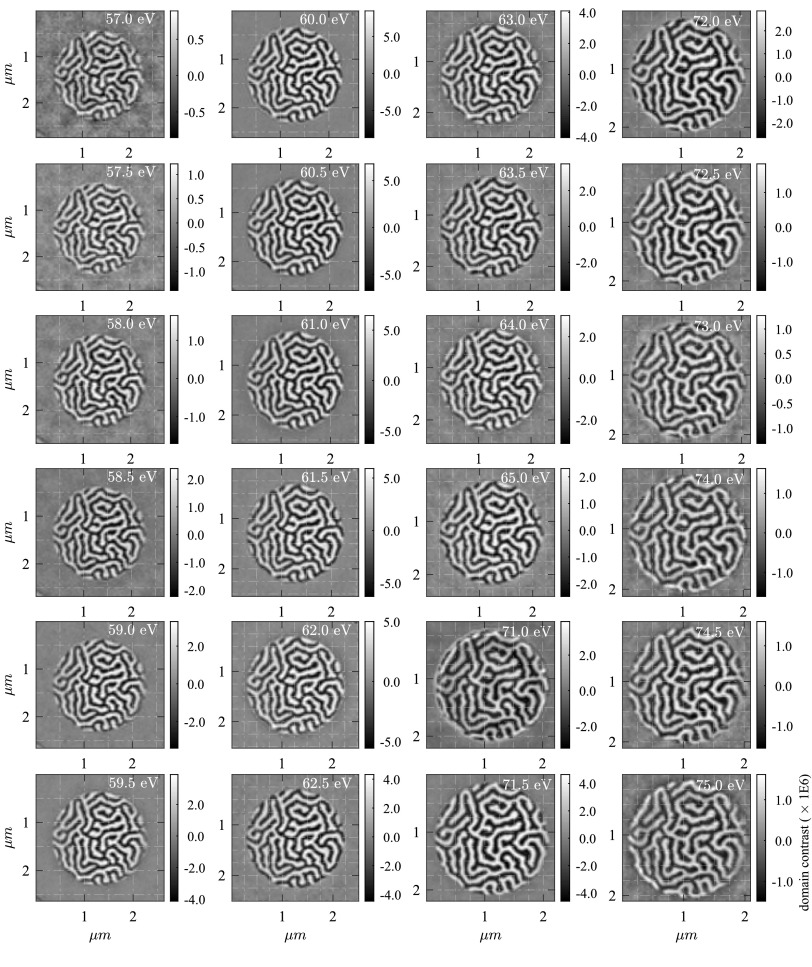
Reconstructions of the magnetic domain network measured for XUV photon energies from 57.0 eV to 76 eV. High-resolution real-space images of the domain network are reconstructed for the entire energy range. Note that for increasing XUV photon energies (smaller wavelengths), the scaling of the images decreases from 42 nm/pixel at 57 eV to 32 nm/pixels at 75 eV. The contrast at the Pt N_6,7_ edges (>71 eV) is inverted.

The magnetic difference holograms are centered with subpixel accuracy, the sharp edge of the beam stop is blurred by a Gaussian filter, the intensity pattern is transformed to in plane *q*-coordinates^28^ and a 2D Fourier transform yields the real-space reconstructions of the magnetic domain network. Finally, we interpolate the images by increasing the sampling rate by a factor of 4. Assuming a well-defined reference wave, the real and imaginary parts of the reconstructed images allow to deduce the dispersive and absorptive part of the dichroic index of refraction.^29^ However, in the XUV energy range, the wavelengths are on the order of 20 nm and start to approach the size of the reference hole diameters such that the wave guiding effects have to be taken into account. These are expected to exhibit a subtle dependence on the exact shape of the reference hole and on the XUV wavelengths and, hence, will result in an additional and *a priori* unknown reference wave phase shift.^30^ Therefore, we define a measure for the total magnetic contrast as the sum of the real and imaginary part of the reconstructions. In Figure 3, we show the resulting real space images of the magnetic domain network as a function of real space coordinates and for all recorded energies. The black and white regions within the circular field of view correspond to areas of magnetization pointing into opposite out-of-plane directions. The color map is scaled from the minimum to the maximum value within each image. We observe clear and well resolved domain patterns over the entire energy range. Note that the images have the same number of pixels in both spatial dimensions, the size of one pixel in the reconstruction decreases from 41.7 nm at 57 eV to 31.9 nm at 75 eV (without interpolation). The contrast is inverted for energies larger than 71 eV in agreement with measurements showing an opposite sign of the MCD effect between the Co M and Pt N resonances.^21^

**FIG. 4.**
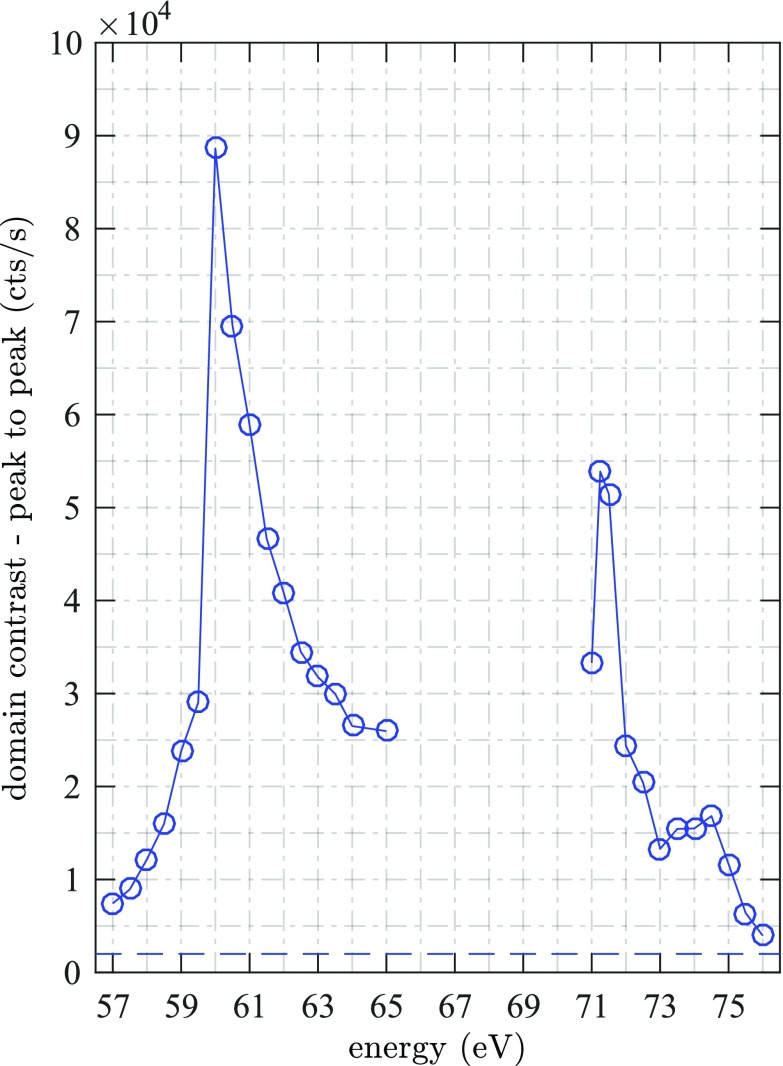
Average peak-to-peak magnetic domain contrast in counts per second as a function of XUV photon energy. We observe a pronounced peak at the Co edge at *E*_Ph_ = 60 eV and two smaller maxima at 74 eV and 71.5 eV which we attribute to core-valence transitions of Pt N_6,7_. The horizontal dashed blue line shows the background signal, i.e., the peak to peak values outside the field of view.

In Figure 4, we show the average peak-to-peak magnetic domain contrast within the field of view normalized to 1 s integration time. At the Co M edge at 60 eV, we observe a pronounced maximum, smaller maxima can be assigned to the N_6_ and N_7_ edge of Pt at 73 eV and 71.5 eV, respectively. We determine the noise level of approximately 10^3^ cts/s (dashed line in Fig. 4) by calculating the average peak to peak value outside the object hole. Note that even at the extreme ends of the energy range, at 57 eV and 76 eV, we can detect high contrast domain patterns, with signal to noise ratios exceeding 3.5 and 2, respectively. The energy dependent contrast variations are in qualitative agreement with the small-angle scattering intensity shown in Fig. 2(b). We attribute the quantitative differences to slightly different properties of the Co/Pt interfaces of the imaging sample (Fig. 1(h)), which are known to sensitively influence the magnitude of the induced magnetization of Pt. The fact that our step width of 0.5 eV undersamples the spectrally narrow Pt N_6_ edge may also cause further quantitative differences.

**FIG. 5.**
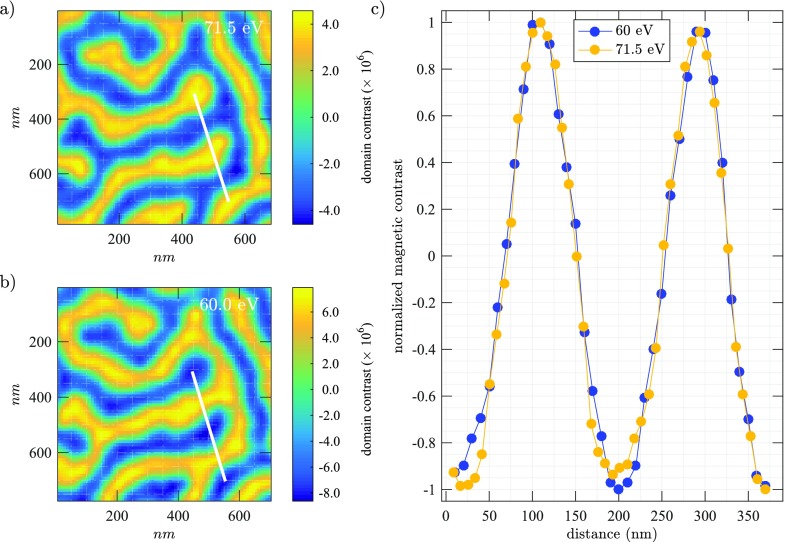
(a) Detail of the reconstructed magnetic domains (a) at 71.5 eV and (b) at 60.0 eV as a function of real space coordinates. (c) Normalized lineouts along the white lines shown in (a) and (b). We observe an inverted, yet identical magnetic domain pattern for Co and Pt layers. The spatial resolution is estimated to be below 80 nm. The solid line is a guide to the eye.

In Figure 5, we present a detail of the measured domain contrast at 71.5 eV and 60 eV. The images are scaled to their actual real-space dimension in nanometer, due to the shorter wavelength one pixel in the reconstruction at 71.5 eV corresponds to 33.6 nm and at 60 eV to 40.0 nm respectively (after interpolation, these numbers reduce to 10 nm and 8.4 nm). In Figure 5(c), we show the normalized line profiles calculated along the white line shown in (a) and (b) and corrected for the inverted contrast. Because in our FTH experiment, the spatial resolution is determined by the reference hole geometry, rather than by the numerical aperture of the setup and wavelength, both measurements have the same resolution on the order of 80 nm. An exact determination of the spatial resolution based on these magnetic images is challenging because on the one hand, a slight high pass-filtering is present due to the use of a central beam stop and because a finite size of the domain wall width has to be taken into account.^15^

The analysis in Figure 5(c) allows an element specific comparison of the lateral magnetization profile with a high spatial resolution. For the investigated Co/Pt multilayer, we observe an identical magnetic domain pattern, indicating a laterally homogeneous magnetization throughout the entire film thickness. In heterostructures or bilayers of Co/Pt, the equilibrium magnetization of Pt is strictly confined to the vicinity of the boundary^19,24^ and the properties of the interface magnetism are governed by spin-orbit interaction. Here, we expect an optically or electrically induced spin injection to lead to a transient spatial rearrangement of magnetic order at the Co/Pt boundary. This makes multi-color coherent imaging experiments a unique experimental tool to study the element-specific response of magnetization dynamics in three-dimensional space and promises to shed light on a wealth of intriguing Co/Pt interface phenomena.^1–9^

## IV. SIMULTANEOUS TWO-COLOR COHERENT IMAGING

In the following, we present the first experimental realization of a coherent imaging experiment with direct and simultaneous access to the element specific and spatially resolved magnetization of two distinct elements, Co and Pt.

The experiment was carried out at the free electron laser (FEL) facility FERMI delivering brilliant, femtosecond pulses in the XUV spectral region.^31,32^ Briefly, FERMI relies on a seeded harmonic scheme where the FEL emission occurs at a harmonic of an external UV seed pulse. In a first undulator, the seed interacts with a bunch of relativistic electrons and modulates their energy longitudinally with the periodicity of the seed wavelength, λ_Seed_. Then, in a magnetic chicane, electrons follow an energy-dependent path which converts the energy modulation into an electron density modulation, forming micro-bunches that emit coherently in a second undulator section, tuned to the desired harmonic wavelength.

For simultaneous probing of the magnetizations of Co and Pt, a two color operation of the FEL is required. For this purpose, the second undulator was split into two subsections, being resonant at λ_FEL,1_ = λ_Seed,1_/*m* and λ_FEL,2_ = λ_Seed,1_/*n*, where *n* and *m* are the integer harmonic numbers. In such a configuration, the two colors are synchronized and probe the sample simultaneously. More advanced generation schemes may be used for introducing a delay of few hundreds of femtoseconds between the two colors,^12,13,33,34^ while a XUV-split-and-delay scheme offers a complete control over the temporal separation and spatial overlap of the probe pulses, at the cost of a more complex experimental implementation.

**FIG. 6.**
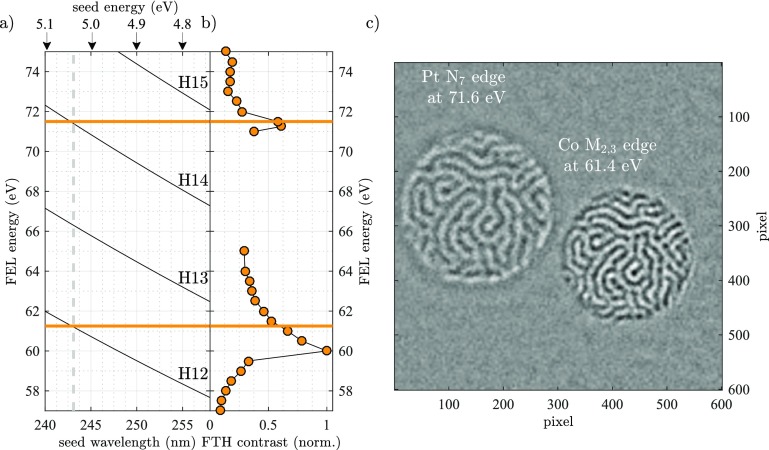
(a), (b) FEL energy as a function of seed wavelength shown for harmonics H12 to H15. For a single seed wavelength of λ_SEED_ = 242.2 nm, we can maximize the magnetic domain contrast at the Pt N_7_ edge at 71.6 eV (H14) and simultaneously get a comparable signal for H12 at 61.4 eV at the Co M_2,3_ edge. (c) Element specific magnetic domain patterns for Pt and Co reconstructed from a single difference hologram. The respective element specific real space information for Co and Pt does not overlap. Note that the pixels correspond to different real-space coordinates due to the different XUV wavelengths employed.

The constraint for the accessible FEL wavelength separation is shown in Figure 6(a) and is given by the photon energy of the UV seed laser or multiples of it, i.e., multiples of approximately 5 eV. We replot the energy dependent peak to peak magnetic domain contrast of Figure 3 and indicate the optimal FEL wavelengths by solid orange lines in Figures 6(a) and 6(b). A comparable magnetic contrast for Pt and Co is obtained at 71.6 eV (λ_FEL,2_ = 17.3 nm) and 61.4 eV (λ_FEL,1_ = 20.2 nm), respectively.

For the two-color imaging experiment, we adapted the FTH mask geometry to avoid a spatial overlap of the reconstructed objects. In the reconstruction of the (n×n)-sized hologram, the position of the object is wavelength dependent and is given by^35^

$$\vec{x}\left(\lambda_{FEL}\right)=-\vec{r}/r\cdot nd_{pix}r/\left(\lambda_{FEL}D_{FTH}\right),$$ (2)

in units of pixels (cf. Fig. 1(f)). The vector connecting the reference and object hole is denoted by $\vec{r}$. Hence, spatially separated images of the $d_{r}=2$ *μ*m sized object at the two different FEL wavelengths are achieved by adding two additional reference holes at a larger distance of $r=|\vec{r}|$ = 13 *μ*m. With such multi-reference FTH imaging,^36^ additional care has to be taken, that none of the cross correlations between the various reference holes overlap with the object-reference correlation of interest.

The experiment was carried out at the end-station DiProI,^37,38^ the geometry of the setup as well as the sample (cf. Figs. 1(i) and 1(h), respectively) is identical to the synchrotron measurements described above. We reduce the FEL intensity of the two pulses to 2 ± 0.3 *μ*J at 61.4 eV (Co) and 3 ± 0.3 *μ*J at 71.6 eV (Pt) with a spot size of 180 *μ*m × 190 *μ*m (FWHM) in order to avoid the X-ray induced changes of the domain pattern.^16,39^ This corresponds to approximately 8 × 10^6^ photons/*μ*m^2^/pulse and 10^7^ photons/*μ*m^2^/pulse at the Co and Pt resonance, respectively. At a 10 Hz repetition rate, we acquire 5 images with 600 s integration time for both left and right circular polarization. We repeat the same analysis for the digital image reconstruction as described above and additionally increase the signal to noise ratio by summing the reconstructions from the two new reference holes. The simultaneously measured, element-specific, real-space images of the magnetic domain pattern are presented in Figure 6(c). The magnetic domains are clearly resolved, and the resolution is comparable to the measurements presented in Fig. 3. The reconstruction shows no imaging artefacts and has an excellent suppression of the charge scattering.

In the following, we make a conservative estimate on the number of required XUV photons to perform a two-color imaging experiment with <80 nm resolution. Assuming a reduced object hole size in the dispersion direction of $d_{r}=1$ *μ*m and a relative wavelength difference Δλ/λ∼0.15 (e.g., Pt N_7_ and Co M_2,3_ edges) a spatial separation of the two reconstructed images requires a minimum length of the vector $\;|\vec{r}|$ connecting the reference and the object hole of $r=d_{r}\lambda /\Delta \lambda ∼$6 *μ*m or $r/d_{r}=6$. Focusing to a spot size on the sample of 12 *μ*m (FWHM) to homogenously illuminate the reference and object and maintain the photon flux and integration times of the above describe FEL experiment, we would require approximately 10^10^ photons/s in the two-color XUV beam. With advanced reference schemes like multi-reference geometries^36^ or monolithic zone plate focusing reference structures^40^ the signal noise ratio can be further significantly improved. We thus expect that multi-color imaging experiments will be feasible in the near future with lab-based high-harmonic sources.^41^ We note that by increasing the ratio $r/d_{r}\geq 20$ of the FTH mask, one will even be able to use the entire high harmonic spectrum generated by a λ = 800 nm driver laser without any further wavelength selecting optics.

Spatially and temporally resolved spectroscopy with double- or multi-color XUV probe pulses offers the unique opportunity to simultaneously access the element- or electronic-specific response in a single experiment. This is not only of imminent importance for non-repetitive experiments of stochastic processes^10^ or for very high, destructive excitation densities,^42^ but also for complex multi-component or multiphase systems where the excitation is followed by a complex and ultrafast interaction between different constituent elements or different electronic states. Some recent and prominent examples include competing phases in correlated materials showing metal to insulator transitions^43–46^ and chemical inhomogeneities in ferrimagnets exhibiting all-optical switching.^47^

## V. CONCLUSION

We have demonstrated spatially resolved access to element-specific magnetization in Co/Pt heterostructures, both in reciprocal space via SAXS and in real space via FTH. The XMCD effect at the Co M_2,3_ edge as well as the very strong dichroic signals at the O_2,3_ and N_6,7_ edge of Pt give rise to almost background free magnetic scattering signals and lead to bright diffraction peaks in *q*-space and high-contrast and high-resolution magnetic domain images in real space. We presented the first realization of a double-color imaging experiment at the free-electron laser facility FERMI encoding the real space magnetic domain patterns of Co and Pt in a single hologram. We envision the multi-color, real-space spectroscopy at FEL and HHG sources to become a valuable tool to unravel ultrafast interactions within the electronic and spin structure of complex multicomponent and multiphase materials.
